# Effects of monobutyrin and tributyrin on liver lipid profile, caecal microbiota composition and SCFA in high-fat diet-fed rats

**DOI:** 10.1017/jns.2017.54

**Published:** 2017-10-11

**Authors:** Thao Duy Nguyen, Olena Prykhodko, Frida Fåk Hållenius, Margareta Nyman

**Affiliations:** Food for Health Science Centre, Lund University, PO Box 124, SE-221 00, Lund, Sweden

**Keywords:** SCFA, Succinic acid, Gut microbiota, Liver cholesterol, Bile acid-related genes, HFC, high-fat control, LBP, lipopolysaccharide-binding protein, LF, low-fat, LPS, lipopolysaccharide, MB, monobutyrin, TB, tributyrin

## Abstract

Butyric acid has been shown to have suppressive effects on inflammation and diseases related to the intestinal tract. The aim of the present study was to investigate whether supplementation of two glycerol esters, monobutyrin (MB) and tributyrin (TB), would reach the hindgut of rats, thus having an effect on the caecal profile of SCFA, microbiota composition and some risk markers associated with chronic inflammation. For this purpose, rats were fed high-fat diets after adding MB (1 and 5 g/kg) and TB (5 g/kg) to a diet without any supplementation (high-fat control; HFC). A low-fat (LF) diet was also included. In the liver, total cholesterol concentrations, LDL-cholesterol concentrations, LDL:HDL ratio, and succinic acid concentrations were reduced in rats given the MB and TB (5 g/kg) diets, compared with the group fed the HFC diet. These effects were more pronounced in MB than TB groups as also expressed by down-regulation of the gene *Cyp8b1*. The composition of the caecal microbiota in rats fed MB and TB was separated from the group fed the HFC diet, and also the LF diet, as evidenced by the absence of the phylum TM7 and reduced abundance of the genera *Dorea* (similar to LF-fed rats) and *rc4-4*. Notably, the caecal abundance of *Mucispirillum* was markedly increased in the MB group compared with the HFC group. The results suggest that dietary supplementation of MB and TB can be used to counteract disturbances associated with a HFC diet, by altering the gut microbiota, and decreasing liver lipids and succinic acid concentrations.

During recent years, it has become more and more evident that the colonic environment is of crucial importance for both colonic and metabolic health. For instance, inflammation, which is a common denominator observed in the onset of diseases, is mediated via the colon by microbiota products. A diet high in fat is thought to have a direct effect on obesity-related inflammation by altering the gut microbiota composition and increasing intestinal permeability, together with increased lipopolysaccharide (LPS) release into the systemic circulation^(^^1^^–^^3^^)^. Lipopolysaccharide-binding protein (LBP) binds to LPS, and has been suggested as a biomarker of LPS^(^^4^^,^^5^^)^. Especially, a high fat intake changes the SCFA formation to lower proportions of butyric acid and higher of succinic acid, an intermediary colonic metabolite classically known as a precursor of propionic acid^(^^6^^)^, as shown in rats in several studies^(^^7^^,^^8^^)^. Furthermore, high amounts of succinic acid may be formed at a low bacterial activity in the colon caused by antibiotic treatment^(^^9^^)^; and succinic acid concentration has been shown to correlate with the severity of caecal inflammation in a mouse model of ulcerative colitis^(^^10^^)^. Taken together, an impaired gut health initiated by an imbalanced microbiota composition can be the origin of both colonic and metabolic diseases, apparently highlighting a protective role of SCFA.

SCFA are the main products of fermentation of indigestible carbohydrates by the gut microbiota^(^^11^^)^. Besides their traditional role as an energy source for colonocytes, SCFA are well known for their potent anti-inflammatory function. For example, butyrate has been shown to suppress production of pro-inflammatory molecules via inhibition of inflammatory pathways^(^^12^^,^^13^^)^. The importance of butyrate for maintaining colonic health has been shown in a number of studies in both human subjects and rats with butyrylated resistant starch (high-amylose starch acetylated with butyrate), which delivers butyric acid in physiologically effective concentrations to the colon^(^^14^^–^^17^^)^. For example, in the study by Furusawa *et al.*^(^^15^^)^, butyrylated starch was shown to be more effective than the high-amylose starch *per se* to regulate the differentiation of T cells in the colon and to suppress inflammatory and allergy responses which were connected with decreased colitis induced in mice. Application of butyric acid alone as a therapeutic agent is hindered due to difficulties in rapid metabolism, very short half-life and strong odour of butyrate, making direct oral consumption unacceptable. To overcome this drawback, there is a growing interest in developing SCFA derivatives with stable properties until transported to the targeted organs. The most well-known derivative is tributyrin (TB), a pro-drug of butyrate, which has been shown to reduce release of pro-inflammatory cytokines^(^^18^^)^, induce growth inhibition and apoptosis of colon cancer cells, even more effectively than butyrate^(^^19^^)^. The effectiveness of TB has also been evaluated in rat models of colon^(^^20^^)^ and liver cancer^(^^21^^,^^22^^)^.

In the present study, two SCFA-based ester products, monobutyrin (MB) and TB, were mixed into high-fat control (HFC) diets and given to conventional rats for 3 weeks. A high-fat setting was used to provoke a low formation of butyrate and a more inflammatory environment in the colon, as previously reported^(^^8^^)^ in the same rat model. The aim was to investigate whether these esters would reach the colon and affect the colonic properties of some metabolic risk-markers associated with chronic inflammation of the gut. For this purpose, the caecal content of SCFA and microbiota composition were analysed in the rats. Furthermore, the lipid profile (total cholesterol, LDL-cholesterol, HDL-cholesterol and TAG) and SCFA in the liver and serum were examined as well as serum LBP. Succinic acid and the expression of bile acid-related primary genes in the liver (*Cyp7a1*, *Cyp8b1* and *Nr0b2*) were also determined.

## Materials and methods

### Diets

MB and TB were kindly provided by Perstorp AB. To induce a pre-inflammatory state in the colon concerning the microbiota composition and gut metabolites (SCFA and succinic acid), all test diets containing the butyrins were prepared in a high-fat setting. A high-fat diet without any butyrins was used as a control (HFC) and a low-fat (LF) diet was also included as a reference. All test diets were prepared according to the composition in Table 1. Casein was used as the protein source, whereas cellulose was chosen as the fibre source since it is resistant to fermentation in the gut (humans and rats) and therefore gives low amounts of SCFA^(^^23^^)^. Wheat starch was used to adjust the DM content. The type of wheat starch used in the present study has been previously shown to be completely digested and does not contribute to subsequent formation of any SCFA in the caecum or in the bloodstream of rats^(^^24^^)^.

**Table 1.** Composition (g/kg dry weight) of the test diets

Diet…	LF	HFC	0·1 MB	MB	TB
Components
Casein*	150·0	150·0	150·0	150·0	150·0
dl-Methionine*	1·2	1·2	1·2	1·2	1·2
Butter†	0·0	180·0	180·0	180·0	180·0
Rapeseed oil	50·0	50·0	50·0	50·0	50·0
Sucrose	100·0	100·0	100·0	100·0	100·0
Cellulose‡	50·0	50·0	50·0	50·0	50·0
Mineral mixture§	48·0	48·0	48·0	48·0	48·0
Vitamin mixture‖	8·0	8·0	8·0	8·0	8·0
Choline chloride*	2·0	2·0	2·0	2·0	2·0
Ester product	0·0	0·0	1·0	5·0	5·0
Wheat starch¶	590·8	410·8	409·8	405·8	405·8

LF, low-fat; HFC, high-fat control; 0·1 MB, monobutyrin (1 g/kg diet); MB, monobutyrin (5 g/kg diet); TB, tributyrin (5 g/kg diet).

* Sigma-Aldrich.

† Arla Foods.

‡ FMC BioPolymer.

§ Containing (g/kg): 0·37 CuSO_4_.5H_2_O, 1·4 ZnSO_4_.7H_2_O, 332·1 KH_2_PO_4_, 171·8 NaH_2_PO_4_.2H_2_O, 324·4 CaCO_3_, 0·068 KI, 57·2 MgSO_4_, 7·7 FeSO_4_.7H_2_O, 3·4 MnSO_4_.H_2_O, 0·02 CoCl.6H_2_O, 101·7 NaCl, 0·019 chromium (III) chloride and 0·011 sodium selenite (Lantmännen).

‖ Containing (g/kg): 0·62 menadione, 2·5 thiamin hydrochloride, 2·5 riboflavin, 1·25 pyridoxine hydrochloride, 6·25 calcium pantothenate, 6·25 nicotinic acid, 0·25 folic acid, 12·5 inositol, 1·25 *p*-aminobenzoic acid, 0·05 biotin, 0·00375 cyanocobalamin, 0·187 retinyl palmitate, 0·00613 calciferol, 25 d-*α*-tocopheryl acetate, 941·25 maize starch (Lantmännen).

¶ Cargill; varied depending on the ester and fat content of the test diets.

The study design resulted in five diets, including three test diets containing glycerol esters. The ester products were added in a concentration of 5 g/kg in the diets calculated on a dry weight basis. These diets were abbreviated as MB or TB. In addition, a diet with 1 g/kg MB was included to get an idea about the dose-dependent effect of this product. This diet is abbreviated as 0·1 MB. The doses chosen were based on results from a pilot study and from previous work^(^^25^^)^. The composition of MB and TB is shown in Supplementary Table S1.

### Animals and experimental design

The present study was conducted based on guidelines for the protection of laboratory animals used for scientific purposes, and it was approved by the Local Ethical Review Committee for animal experiments in Lund, Sweden (approval number M 295-12).

Male rats, *Rattus norvegicus*, of Wistar strain (Taconic) with an initial average weight of 142 (sem 8) g, were randomly divided into five groups of seven. Each group was housed into two cages containing three or four rats per cage. After 3 d acclimatisation to the environment (21°C, 12 h light–12 h dark cycle), they were given the test diets. The experiment was performed in three consecutive weeks in which the animals were allowed to have free access to the test diets and water placed on the cage lid. New feed was added every 2–3 d, while residues were weighed and recorded. Rat body weights were recorded every week.

After the experimental period, the rats were anaesthetised for tissue harvesting by a subcutaneous injection mixture (1:1:2) of Hypnorm (Division of Janssen-Cilag Limited, Janssen Pharmaceutica), Dormicum (F. Hoffmann-La Roche AG) and autoclaved Milli-Q Millipore water, at a dose of 0·15 ml/100 g body weight. Rats from two different groups were randomly chosen for dissection on the same day. The rats were fed with the actual diets until dissection. Blood samples were collected from the hepatic portal vein and placed immediately in serum tubes (SST™ II Advance, Plus Blood Collection Tubes, BD Vacutainer). The serum samples, obtained after centrifugations, were stored at −40°C until analysis of SCFA, LBP, cholesterol and TAG. The caecum was removed and weighed with and without its content. Caecal tissue was washed with Milli-Q Millipore water, while content was subjected to pH measurement before being stored at −20°C until analysis of SCFA and gut microbiota composition. Other organs including the liver, spleen, stomach, small intestine and colon were removed, weighed and stored at −80°C for further analysis. SCFA and succinic acid were analysed in the liver. Blood glucose was measured immediately by a HemoCue^®^ Glucose 201^+^ Analyzer (HemoCue AB) after the collection of blood from the hepatic portal vein.

### Analyses

#### Carboxylic acids

SCFA in caecal content were extracted with acidified water before being measured by a methodology using direct injection GC^(^^26^^)^. Concentrations of SCFA in serum samples were pre-enriched and extracted by hollow fibre before being injected and analysed with GC^(^^27^^)^.

SCFA together with succinic acid were also analysed in freeze-dried liver samples. For SCFA, the same method as for caecal content was applied^(^^26^^)^. Succinic acid was measured by ion-exclusion chromatography with a method developed at the department (MIC-2 Advanced modular IC; Metrohm AG). Approximately, 100 mg of liver samples were diluted with Milli-Q Millipore water, centrifuged at 4000 rpm for 20 min, filtered through a 0·45 mm syringe filter, and injected into a Metrosep organic column (250 × 7·8 mm, Metrohm). The column oven temperature was 70°C and the effluent (0·5 mm-H_2_SO_4_) flow rate was 0·6 ml/min. The suppressor was regenerated using a solution of 10 mm-LiCl, followed by Milli-Q Millipore water. The analysis time for each run lasted for 25 min.

#### Cholesterol and TAG in blood and liver

Freeze-dried liver samples were used for the determination of total cholesterol, HDL- cholesterol, LDL-cholesterol and TAG. Lipids were extracted using a modified method with low-toxicity solvent^(^^28^^)^. Briefly, 20 mg were dissolved in a 3:2 solution containing hexane (Sigma-Aldrich), isopropanol (Merck), with 0·005 % (v/w) 2,6-di-tert-butyl-4-metylphenol (Merck) before being centrifuged, and the supernatants were evaporated under N_2_ flow at room temperature. The dried lipid extracts were re-dissolved in 1 ml of isopropanol containing 1 % (v/v) Triton X100 (Sigma-Aldrich). Total cholesterol and TAG in liver and serum were determined spectrophotometrically using Infinity Cholesterol/Triglyceride Liquid Stable Reagent (Thermo Scientific), while liver HDL and LDL were measured using the reagents HDL-Cholesterol Plus and LDL-Cholesterol (Thermo Scientific).

#### Lipopolysaccharide-binding protein

Levels of LBP in serum were determined using an LBP ELISA kit for a wide variety of species and the protocol was supplied by the manufacturer (Hycult Biotech).

#### Caecal microbiota

Extraction of DNA was performed from 50–100 mg of rat caecal content using the QIAamp Fast DNA Stool Mini Kit (Qiagen), according to the manufacturer's protocol using an additional bead-beating step. Measurement of DNA concentration was performed using a Qubit 2.0 Fluorometer (Life Technologies). See Supplementary material for further details.

#### Gene expression

PrimePCR for *Nr0b1*, *Cyp7a1* and *Cyp8b1* was purchased from Bio-Rad. See Supplementary material for further details.

### Statistical evaluation

Minitab statistical software (version 17.2.1; AutoBVT Microsoft) was used to evaluate significant differences between groups. Each sample from all analyses was performed in duplicate, except in the gene expression experiment where every sample was tested in triplicate. Grubbs’ test was used to check normality of the data. If the data were not normally distributed, differences between groups were identified after Box–Cox transformation or using the non-parametric Kruskall–Wallis test. One-way ANOVA was applied to determine differences in means between groups. After ANOVA, Dunnett's method was applied to specifically identify which groups, including the group fed the LF diet, were significantly different from the HFC group, to evaluate whether the butyrins could counteract the adverse effects induced by high fat feeding. Furthermore, high-fat butyrin diets were also compared with the LF diet, to confirm the effects of the butyrins. For microbiota analysis, the same statistical approach (from GraphPad Prism 7) was used to find differences in relative abundances, at phylum and genus levels, and to correct for multiple comparisons. Pearson's correlation was used to evaluate association between parametric variables, while Spearman's correlation was applied for non-parametric data. Partial least-squares-projection-to-latent-structures-discriminant analysis (PLS-DA) from SIMCA software (version 14; Umetrics) was used to analyse and visualise how the gut microbiota data at genus level varied in relation to the liver lipids. The confidence level in all statistical tests was specified at 95 %. *P* values <0·05 were statistically significant, while 0·1 ≥ *P* values ≥0·05 were considered as tendency. Results are presented as means with their standard errors.

## Results

### Body weight gain, tissue weights and pH

All rats appeared healthy and active, and gained weight gradually throughout the experiment (Table 2). At the end of the experiment, rats given the MB diet had a 7 % lower final body weight compared with those fed the HFC diet (*P* = 0·024). Body weight gain was similar between all groups fed high-fat diets. The LF diet group had a higher body weight gain than the rats fed the HF diets, but by taking the feed intake into account (feed efficiency ratio) the body weight gain was similar for all groups (0·29–0·33). Caecal pH was between 7·4 and 7·7.

**Table 2.** Final body weight, body weight gain, total feed intake, feed efficiency ratio (body weight gain/feed intake), wet and freeze-dried liver weight, spleen weight, caecal tissue, caecal content and caecal pH in rats fed a low-fat (LF) diet, a high-fat control (HFC) diet or the HFC diet supplemented with monobutyrin at 1 g/kg diet (0·1 MB), MB at 5 g/kg diet (MB) or tributyrin at 5 g/kg diet (TB) for 21 d (Mean values with their standard errors; *n* 7)

Group…	LF		HFC		0·1 MB		MB		TB	
Parameters	Mean	sem	Mean	sem	Mean	sem	Mean	sem	Mean	sem
Final body weight (g)	292	8	270	4	275	7	252*†††	7	256††	4
Body weight gain (g)	137	6	120	4	129	7	113†	6	116†	4
Total feed intake (g/rat)	453	2	417	2	389	10	347	9	355	2
Feed efficiency ratio (g/g feed)	0·30	0·01	0·29	0·01	0·33	0·02	0·33	0·02	0·33	0·01
Wet liver weight (g)	11	0·4	10	0·4	10	0·3	9†	0·3	10†	0·2
Freeze-dried liver weight (g)	3·3	0·2	3·1	0·1	3·1	0·1	2·8†	0·1	2·9	0·1
Spleen weight (g)	0·6	0·03	0·6	0·03	0·6	0·02	0·5	0·03	0·6	0·03
Caecal tissue weight (g)	0·5	0·02	0·5	0·02	0·5	0·03	0·6	0·08	0·5	0·02
Caecal content weight (g)	1·7	0·2	2·2	0·3	2·1	0·3	1·9	0·2	1·7	0·2
Caecal pH	7·4	0·1	7·5	0·1	7·5	0·1	7·7	0·3	7·6	0·3

* Mean value was significantly different from that of the HFC group (*P* < 0·05; one-way ANOVA and Dunnett's test).

Mean value was significantly different from that of the LF group: † *P* < 0·05, †† *P* < 0·01, ††† *P* < 0·001 (one-way ANOVA and Dunnett's test).

No difference was found in absolute weights of the liver, spleen and caecal tissue.

### Carboxylic acids

#### Caecum

Addition of TB to the HFC diet reduced total caecal amounts of SCFA from 82 to 56 µmol (Table 3; *P* = 0·027). The amount of individual SCFA was also lower and these values were significant for all acids except butyric acid and isobutyric acid (acetic acid (*P* = 0·051), propionic acid (*P* < 0·0001), valeric acid (*P* = 0·024) and isovaleric acid (*P* = 0·027)). Rats in the MB group showed a significant decrease in propionic acid and isovaleric acid (*P* = 0·02 and *P* = 0·03, respectively) compared with the HFC group. Furthermore, both MB and TB groups had a significantly higher ratio of acetic acid:propionic acid compared with the HFC group (*P* = 0·02 and *P* = 0·008, respectively) (Supplementary Fig. S1). No difference in the ratios of acetic acid:butyric acid or acetic acid:propionic plus butyric acid between the test groups and the HFC group could be seen. The proportion of propionic acid was significantly lower in the MB and TB groups (11 %) compared with the HFC group (13 %; *P* < 0·0001; Supplementary Table S2). The SCFA profile of the 0·1 MB group did not differ from that of the HFC group.

**Table 3.** SCFA in the caecum, portal serum, and liver of rats fed a low-fat (LF) diet, a high-fat control (HFC) diet or the HFC diet supplemented with monobutyrin at 1 g/kg diet (0·1 MB), MB at 5 g/kg diet (MB) or tributyrin at 5 g/kg diet (TB) for 21 d (Mean values with their standard errors; *n* 7)

Group…	LF		HFC		0·1 MB		MB		TB	
Locations	Mean	sem	Mean	sem	Mean	sem	Mean	sem	Mean	sem
Caecal pool (μmol)										
Total	100	11	82	10	85	5	65†	6	56*††	7
Acetic	71	8	57	7	60	4	48†	5	41††	5
Propionic	16	2	11†	1	12	1	8*††††	1	6***††††	1
Butyric	9	2	9	1	8	1	8	1	6	1
Valeric	1·6	0·2	1·5	0·2	1·6	0·1	1·2	0·1	1·0*†	0·1
Isobutyric	1·6	0·2	1·2	0·3	1·2	0·1	0·7††	0·1	0·8†	0·2
Isovaleric	1·32	0·2	1·35	0·2	1·22	0·1	0·81*	0·1	0·88	0·1
Portal serum concentration (μmol/l)										
Total	743	32	753	80	822	63	693	46	632	46
Acetic	628	35	635	64	712	54	575	30	540	41
Propionic	47	2	45	6	42	4	28*††	4	21**†††	1
Butyric	32	2	38	6	36	6	58	13	47	5
Valeric	9	2	13	2	13	2	17	2	14	2
Isobutyric	14	1	13	1	11	1	8*†	1	8*†	1
Isovaleric	15	3	11	2	8	2	8	1	7	1
Liver (μmol)										
Total	304****	33	147††††	25	144††††	12	127††††	8	186††	16
Acetic	288****	31	136††††	24	136††††	11	113††††	8	171†††	15
Propionic	4·7	0·4	3·7	0·5	3·2†	0·4	3·1†	0·2	4·3	0·2
Butyric	2·8*	0·3	2·1†	0·1	1·6†††	0·3	2·0†	0·1	2·1	0·1
Valeric	1·1	0·2	0·5	0·1	0·5	0·1	0·6	0·1	0·6	0·1
Isovaleric	9·1	3·1	4·9	1·5	3·3	0·8	8·0	1·0	7·1	1·0

Mean value was significantly different from that of the HFC group: * *P* < 0·05, ** *P* < 0·01, *** *P* < 0·001, **** *P* < 0·0001 (one-way ANOVA and Dunnett's test).

Mean value was significantly different from that of the LF group: † *P* < 0·05, †† *P* < 0·01, ††† *P* < 0·001, †††† *P* < 0·0001 (one-way ANOVA and Dunnett's test).

The total and specific SCFA were generally lower with groups fed high-fat diets compared with the group fed the LF diet, and reach significance for the MB and TB groups in most cases (*P* < 0·0001 to *P* < 0·05). An exception was butyric acid that was similar in all groups.

#### Serum

Rats fed the MB and TB diets generally exhibited lower serum concentrations of SCFA compared with those consuming the HFC diet and reached significance for propionic acid (Table 3; *P* = 0·003) and isobutyric acid (*P* = 0·01). Interestingly, butyric acid concentrations were somewhat higher with the MB and TB diets (58 and 47 µmol/l) compared with the HFC diet (38 µmol/l), but the differences were not significant.

There was no difference in serum SCFA between rats fed the LF and HFC diets.

#### Liver

There was no difference in the amount of SCFA in the liver of rats after supplementation of butyrins compared with those fed the HFC diet. However, the TB group tended to have higher concentrations of total SCFA (*P* = 0·093), acetic acid (*P* = 0·074) and propionic acid (*P* = 0·092) compared with the MB group (Table 3). Furthermore, the higher dose of MB exhibited markedly higher amounts of isovaleric acid than the 0·1 MB group (*P* = 0·024). No difference in the amount of valeric acid was found between any of the groups. The LF group had higher total liver SCFA, acetic acid and butyric acid than the HFC group (*P* < 0·05).

**Fig. 1.**
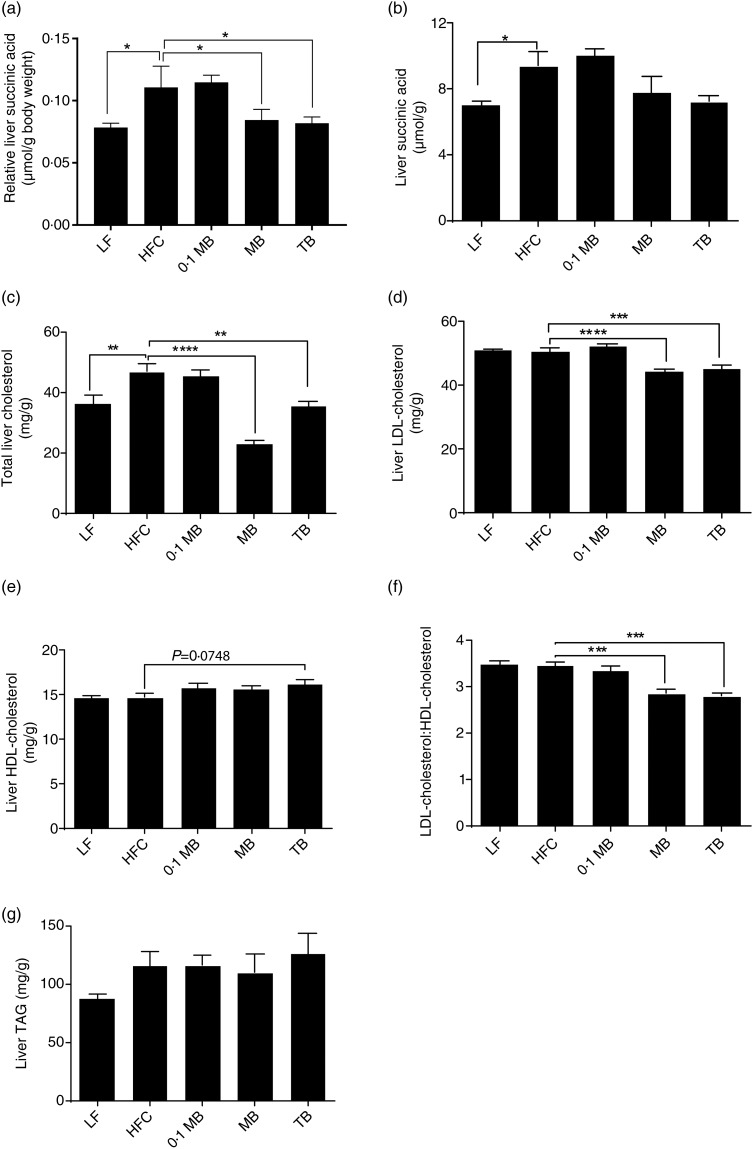
Liver succinic acid and lipid concentrations of rats fed a low-fat (LF) diet, a high-fat control (HFC) diet or the HFC diet supplemented with monobutyrin at 1 g/kg diet (0·1 MB), MB at 5 g/kg diet (MB) or tributyrin at 5 g/kg diet (TB) for 21 d (*n* 7/group). (a) Relative succinic acid (total amount of succinic acid in the liver/final body weight, μmol/g); (b) succinic acid concentration (μmol/g); (c) total cholesterol (mg/g); (d) LDL-cholesterol (mg/g); (e) HDL-cholesterol (mg/g); (f) LDL-cholesterol:HDL-cholesterol ratio; (g) TAG (mg/g). Values are means, with standard errors represented by vertical bars. Mean value was significantly different from that of the HFC group: * *P* < 0·05, ** *P* < 0·01, *** *P* < 0·001, **** *P* < 0·0001 (one-way ANOVA and Dunnett's test).

Regarding succinic acid, there were no differences in the concentration between the high-fat diets (Fig. 1(b)). However, when calculating the amount of succinic acid in relation to body weight it was significantly lower in MB and TB groups than in the HFC group (Fig. 1(a); *P* = 0·033, *P* = 0·023, respectively). Furthermore, the concentration of succinic acid was lower in the group fed the LF diet compared with the group fed the HFC diet (Fig. 1(b); *P* = 0·045), and very similar to the MB and TB groups. The ratio between succinic acid and butyric acid was higher in the group fed the HFC diet than the group fed the LF diet (14·1 *v*. 8·4 µmol/μmol; *P* < 0·05). It decreased when MB and TB was added to the diet, but not significantly.

#### Correlations

**Fig. 2.**
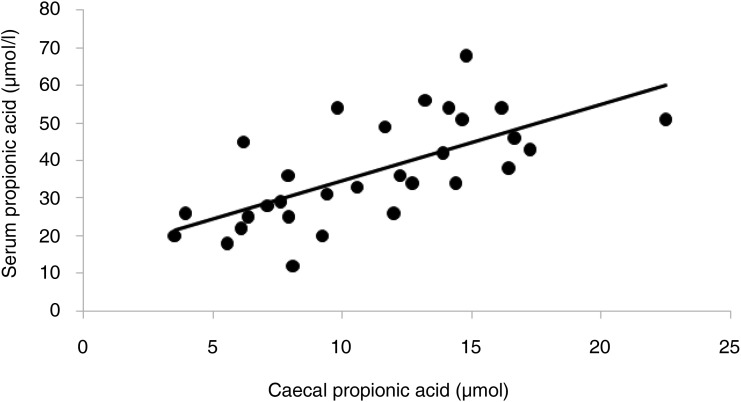
Pearson's correlation of propionic acid in the caecum and portal serum of rats fed a low-fat diet, a high-fat control (HFC) diet or the HFC diet supplemented with monobutyrin at 1 g/kg diet, monobutyrin at 5 g/kg diet or tributyrin at 5 g/kg diet for 21 d (*r* 0·664; *P* < 0·001).

In previous studies, caecal amounts of propionic and butyric acids formed in rats from different fibre-rich food components have been reflected and related to those in portal blood^(^^29^^)^. There was a strong correlation (Fig. 2; *r* 0·664; *P* < 0·001) of caecal propionic acid to those in portal serum also in this study, but not with butyric acid. Furthermore, no correlation was seen between any of the SCFA in the liver and those in the caecum or portal blood, except a tendency for valeric acid to those in portal serum (*r* 0·412; *P* = 0·071).

### Cholesterol and TAG

#### Liver

Incorporation of MB and TB to a HFC diet resulted in an improvement of the liver lipid profile, as evaluated by a significant decrease in hepatic total cholesterol concentrations (Fig. 1(c); *P* < 0·0001 for MB and *P* = 0·0022 for TB, respectively), LDL-cholesterol (Fig. 1(d); *P* < 0·0001, *P* = 0·0005, respectively) and LDL:HDL ratio (Fig. 1(f); *P* = 0·0006, *P* = 0·0002, respectively). The effects on total cholesterol and LDL concentrations were more pronounced in the MB group than in the TB group, as evidenced with 51 and 12 % reductions in total and LDL-cholesterol, respectively, by MB treatment and 24 and 10 % reductions, respectively, by TB treatment. Interestingly, HDL-cholesterol concentrations tended to be higher in the TB group than the HFC group (Fig. 1(e); *P* = 0·0748), which could not be seen with MB. The lipid profile induced by high-fat feeding was not affected by 0·1 MB. TAG concentrations remained similar among all groups (Fig. 1(g)).

The group fed the LF diet had lower total cholesterol concentrations than the group fed the HFC diet (Fig. 1(c); *P* = 0·007). Furthermore, it was similar to the group fed TB but higher than the group fed MB (*P* = 0·0025).

In addition, the total cholesterol and LDL concentrations in the liver were positively correlated with the amount of succinic acid (*r* 0·497, *P* = 0·003 and *r* 0·43, *P* = 0·01, respectively).

#### Serum

**Fig. 3.**
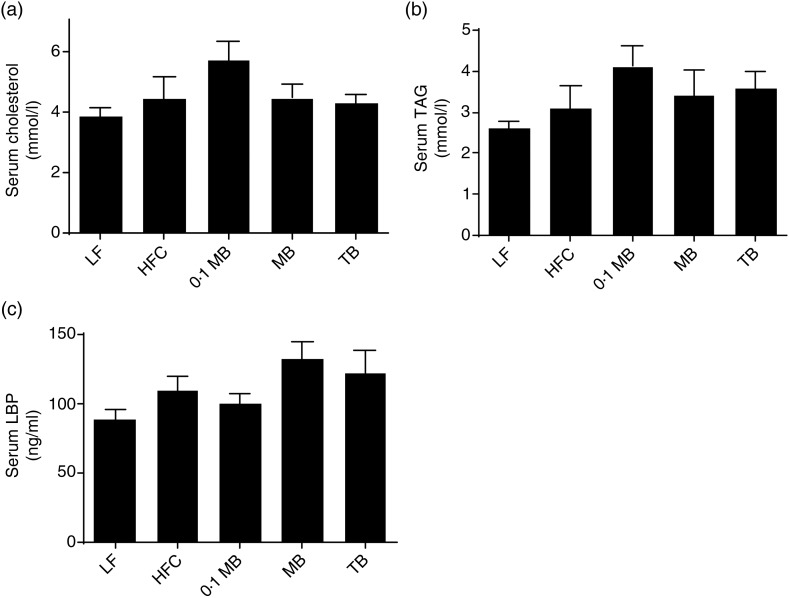
Serum lipid and lipopolysaccharide-binding protein (LBP) concentrations of rats fed a low-fat (LF) diet, a high-fat control (HFC) diet or the HFC diet supplemented with monobutyrin at 1 g/kg diet (0·1 MB), MB at 5 g/kg diet (MB) or tributyrin at 5 g/kg diet (TB) for 21 d (*n* 7/group). (a) Total cholesterol (mmol/l); (b) TAG (mmol/l); (c) LBP (ng/ml). Values are means, with standard errors represented by vertical bars.

None of the added esters caused any changes in serum concentrations of total cholesterol and TAG (Fig. 3(a) and (b)).

### Lipopolysaccharide-binding protein

Concentrations of the inflammatory marker LBP were similar between the test groups and the HFC group (Fig. 3(c)). No significant differences between groups fed the HFC and LF diets could be seen either, although LBP was lower with the LF group.

### Gene expression in the liver

No significant change was observed in the expression of *Cyp7a1* (*P* = 0·12) and *Nr0b2* in the liver between the groups (Table 4). In contrast, relative mRNA expression of *Cyp8b1* in the MB group was lower than in the HFC group (*P* = 0·031), suggesting that a lower amount of bile acids had been formed due to lower available amounts of cholesterol. Moreover, liver cholesterol was positively correlated with the expression of *Cyp8b1* (*r* 0·546; *P* = 0·004), and also *Cyp7a1*, although to a lesser extent (*r* 0·441; *P* = 0·024).

**Table 4.** Relative mRNA expression of *Nr0b2*, *Cyp7a1* and *Cyp8b1* in the liver of rats fed a low-fat (LF) diet, a high-fat control (HFC) diet or the HFC diet supplemented with monobutyrin at 1 g/kg diet (0·1 MB), MB at 5 g/kg diet (MB) or tributyrin at 5 g/kg diet (TB) for 21 d (Mean values with their standard errors; *n* 7, except *n* 6 for HFC)

Group…	LF		HFC		0·1 MB		MB		TB	
Genes	Mean	sem	Mean	sem	Mean	sem	Mean	sem	Mean	sem
*Nr0b2*	2·90	0·04	2·83	0·04	2·85	0·02	2·76	0·06	2·76	0·05
*Cyp7a1*	3·09	0·07	2·92	0·07	2·84	0·08	2·66††	0·09	2·68††	0·07
*Cyp8b1*	2·54	0·04	2·52	0·04	2·51	0·02	2·37*†	0·06	2·42	0·03

* Mean value was significantly different from that of the HFC group (*P* < 0·05; one-way ANOVA and Dunnett's test).

Mean value was significantly different from that of the LF group: † *P* < 0·05, †† *P* < 0·01 (one-way ANOVA and Dunnett's test).

There was no difference in gene expression between groups fed the LF diet and HFC diet.

### Caecal microbiota

**Fig. 4.**
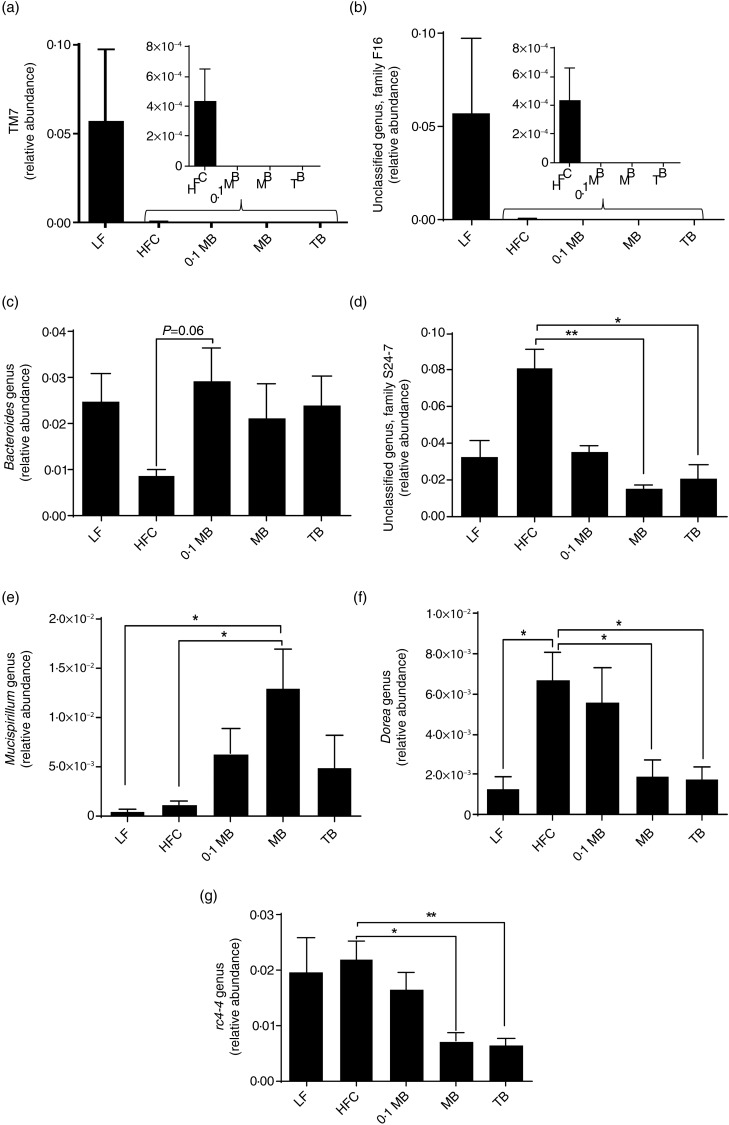
Relative abundance of caecal microbial taxa in rats fed a low-fat (LF) diet, a high-fat control (HFC) diet or the HFC diet supplemented with monobutyrin at 1 g/kg diet (0·1 MB), MB at 5 g/kg diet (MB) or tributyrin at 5 g/kg diet (TB) for 21 d (*n* 5–7/group). (a) TM7 phylum; (b) unclassified genus, family F16; (c) *Bacteroides* genus; (d) unclassified genus, family S24-7; (e) *Mucispirillum* genus; (f) *Dorea* genus; (g) *rc4-4* genus. Values are means, with standard errors represented by vertical bars. Mean value was significantly different from that of the HFC group: * *P* < 0·05, ** *P* < 0·01 (one-way ANOVA and Dunnett's test or Kruskall–Wallis test).

The gut microbiota from all high-fat groups were represented by seven phyla: Firmicutes, Bacteroidetes, Verrucomicrobia, Proteobacteria, Deferribacteres, Candidatus Saccharibacteria (known as TM7) and Tenecurites. No change in relative abundance was seen at phylum level, except for the TM7 phylum that was detected in the HFC diet, but not in any of the groups fed the butyrin diets (*P* = 0·0009; Fig. 4(a)). This difference was concurrently reflected, at genus level, by the absence of an unclassified genus in the family F16 in all the butyrin groups (Fig. 4(b); *P* = 0·0009). Alterations were also observed at genus level within the three phyla Bacteroidetes, Firmicutes and Deferribacteres after supplementation of MB and TB. Originating from the phylum Bacteroidetes, an unclassified genus in the family S24-7 showed a markedly decreased abundance in all test groups (Fig. 4(d); *P* < 0·0001), while the abundance of *Bacteroides* tended to be higher in the 0·1 MB group (Fig. 4(c); *P* = 0·06) compared with the HFC group. Within the phylum Deferribacteres, an increased abundance of *Mucispirillum* was noted in the MB group (Fig. 4(e); *P* = 0·038 compared with the HFC group and *P* = 0·018 compared with the LF group). Interestingly, the abundance of two genera in the phylum Firmicutes, namely *Dorea* and *rc4-4*, was significantly lower in MB and TB groups than in the HFC group (Fig. 4(f), *P* = 0·01; Fig. 4(g), *P* = 0·001).

The abundance of TM7, the unclassified genus in the family F16, *Bacteroides*, the unclassified genus in the family S24-7, *Mucispirillum* and *rc4-4* was similar in the HFC and LF groups, while *Dorea* was lower in the LF group than in the HFC group (*P* < 0·05).

### Multivariate data analysis

**Fig. 5.**
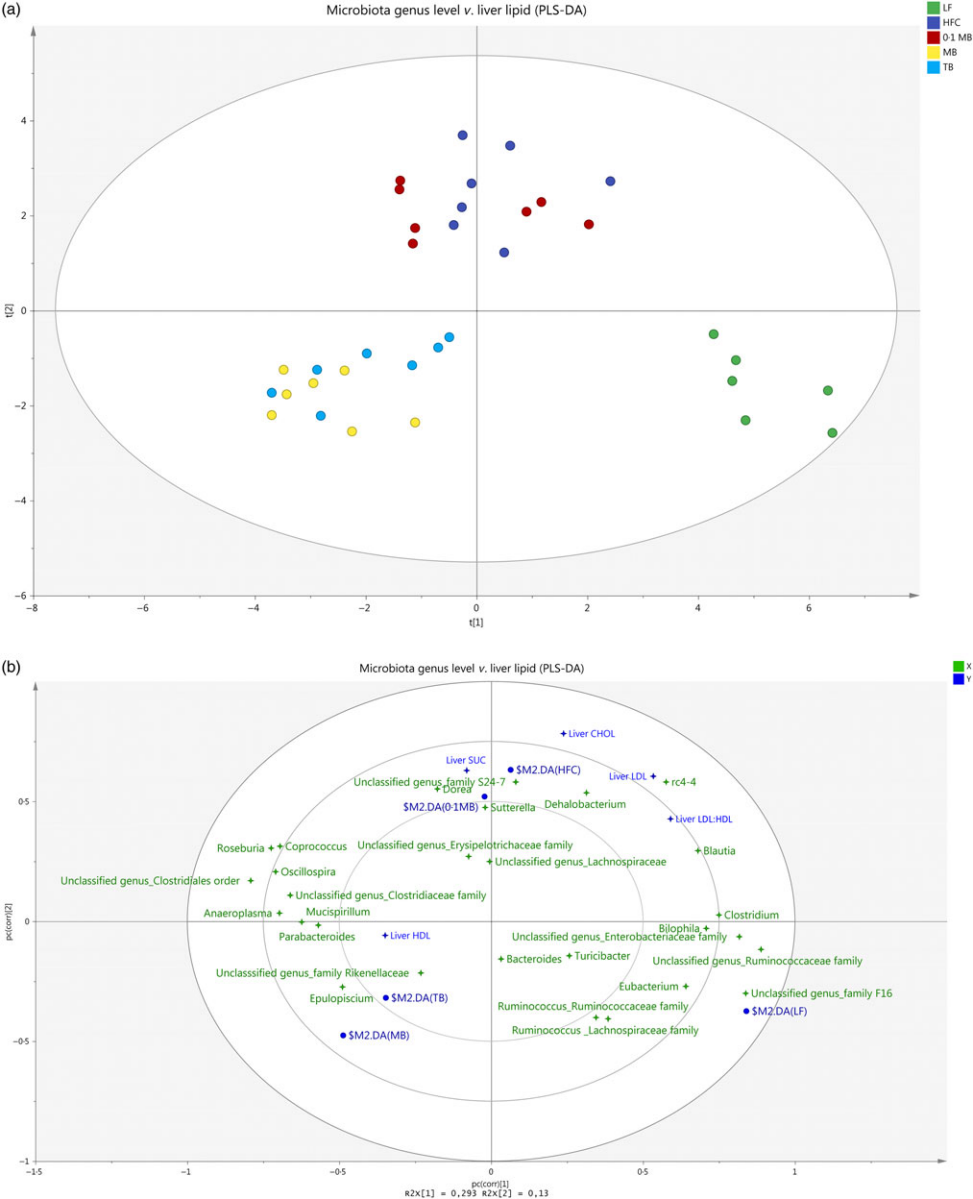
(a) Score scatter plot representing how each group is separated and related to other groups: LF, low-fat diet; HFC, high-fat control diet; 0·1 MB, HFC diet supplemented with monobutyrin at 1 g/kg diet; MB, HFC diet supplemented with monobutyrin at 5 g/kg diet; TB, HFC diet supplemented with tributyrin at 5 g/kg diet. PLS-DA, partial least-squares-projection-to-latent-structures-discriminant analysis. Each circle stands for one rat. (b) Loading scatter plot displays the caecal microbiota composition at the genus level and some liver lipid biomarkers in rats fed the LF diet, the HFC diet or the HFC diet supplemented with 0·1 MB, MB or TB for 21 d (*n* 5–7/group). Variables are marked as four-point stars and groups are showed as circles. CHOL, cholesterol; SUC, succinic acid; X, microbiota genera; Y, liver biomarkers.

A multivariate data analysis was conducted to elucidate how different groups were related to each other concerning the abundance of the microbiota and cholesterol levels (Fig. 5(a)). Treatments with different doses of MB (1 and 5 g/kg) resulted in a clear separation of these groups. Further, animals in the two groups, MB and TB, were clearly separated from the HFC group, as well as from the LF group, whereas the 0·1 MB group was scattered on the same side as the HFC group. Fig. 5(b) represents the gut microbiota composition at the genus level and how these were related to some variables of liver lipids. The HFC group had higher values of liver cholesterol, LDL, LDL:HDL ratio and succinic acid, and was positively related to the abundance of *Dorea* and *rc4-4.* The analytical values with the MB and TB groups were, on the other hand, lower and correlated negatively with these bacterial genera.

## Discussion

This study was performed to test if MB and TB, added to a high-fat diet, could suppress any adverse high fat-induced outcomes, as evaluated by changes in carboxylic acid (SCFA and succinic acid) concentrations, microbiota composition, lipid profile and an inflammatory marker, LBP. The results showed that supplementation of MB and TB, both at 5 g/kg, was effective in decreasing hepatic cholesterol concentrations, while a lower dose of MB (1 g/kg) had no effect. The cholesterol-lowering effect was accompanied by decreased expression of key enzymes involved in bile acid synthesis, and alterations in the microbiota composition with reduced abundance or almost absence of high fat-related inhabitants, but with minor changes in SCFA profile. MB seemed to have more effects than TB which might be due to that more MB on a molar basis was added to the diets.

### Monobutyrin and tributyrin alter the gut microbiota profile

Supplementation of MB and TB to a HFC diet altered the caecal microbiota composition and completely abolished some microbial taxa. The effect was surprisingly obvious at the phylum level, with TM7 present only in the HFC group but totally absent in the MB- and TB-supplemented groups. In terms of counteracting inflammation, this finding is of great interest because higher abundance and diversity of TM7 have been found in inflammatory bowel disease (IBD) patients than in non-IDB subjects^(^^30^^)^. Moreover, TM7 is also involved in oral inflammation where this phylum has been suggested as a modifier shifting the microenvironment, especially in the subgingival plaque, towards a more inflammatory microbiota^(^^31^^)^. Altogether, it could be indicated that both MB and TB had the ability to suppress growth or desirable living conditions of certain bacteria accompanied by high-fat feeding. However, it should be emphasised that the group fed the LF diet had a very high abundance of TM7 compared with the group fed the HFC diet. This may be due to its low content of substrate for the microbiota, microcrystalline cellulose and fat (bile acids), creating a starving environment for the microbiota. A positive control, containing a fermentable fibre component, would perhaps have given more insight into the anti-microbial effects of MB and TB.

### Associations between liver cholesterol and inflammatory markers and microbial taxa induced by monobutyrin and tributyrin

Besides the microbial shift at the phylum level, changes at genus level further revealed a potential link between microbiota and the cholesterol-lowering effect observed in the liver. For instance, the relative abundance of *Dorea* and *rc4-4*, which are generally higher in rodents with diet-induced obesity^(^^32^^)^, was reduced by both the MB and TB supplementations. Furthermore, the abundance of *Dorea* was reduced to similar levels as in the group fed the LF diet, indicating inhibitory effect of esters on this genus. In fact, a positive association has been reported between *Dorea* with serum total cholesterol and LDL in high fat-induced hyperlipidaemic rats^(^^33^^)^, while a decreased abundance of *Dorea* after consumption of β-glucan (yields butyric acid) improves CVD risk factors, lowering TAG levels in mildly hypercholesterolaemic individuals^(^^34^^)^. In contrast to *Dorea*, the genus *Mucispirillum* increased in the MB group compared with the HFC group. *Mucispirillum* is identified as an inhabitant colonising the mucus layer of the gastrointestinal tract of rodents, and may be important to prevent disease development^(^^35^^)^. For instance, under impaired colonic conditions such as colitis in mice, a rapid decrease of this genus during early infection was observed and prolonged until full regeneration of the mucus layer was established^(^^36^^)^. Furthermore, in high-grain-fed goats (a model associated with mucosal injury), an inflammation-suppressing role has been indicated for *Mucispirillum* spp. due to an inverse relationship between its abundance and the expression of IL-12, an inflammatory cytokine^(^^37^^)^. The authors also mentioned that a direct effect of *Mucispirillum* on protecting animals from inflammatory disease should be experimentally tested. However, in the present study, no difference was seen in the concentration of LBP, a potential biomarker of inflammation, which is in contrast to other studies^(^^2^^)^. It may be questioned whether the 3-week feeding of the high-fat diet was long enough to induce a sufficiently high level of low-grade inflammation to be able to detect any anti-inflammatory properties of MB and TB. The higher abundance of *Mucispirillum* associated with butyrin feeding would indicate that supplementation of this ester in sufficient amounts could maintain the intestinal mucus layer from being disrupted by high-fat feeding. Notably, the abundance of *Mucispirillum* was even lower in the group fed the LF diet, indicating that this diet with its low content of substrate for the microbiota is provocative *per se*.

### Monobutyrin and tributyrin induce only minor changes in SCFA profile

The caecal amounts of SCFA with the butyrin diets were lower than expected. Cellulose, added as a fibre component in all the diets, is relatively resistant to bacterial degradation, giving low amounts of SCFA. The SCFA formation with the HFC and LF diets was in concordance with previous studies using the same type of diets, as well as the decrease in SCFA and increase in succinic acid after high fat feeding^(^^8^^,^^32^^,^^38^^)^. However, it could be argued that butyric acid would be detected in higher amounts in the caecum of rats fed the butyrin diets. The reason for the low amounts of butyric acid could be that the butyrins were absorbed very quickly in the caecum of rats or perhaps already in the upper part of the gut. In line with this explanation, it has been shown that TAG are rapidly hydrolysed by gastric^(^^39^^)^ and pancreatic lipase^(^^40^^)^, and readily absorbed into the digestive mucosa in hydrolysed or esterified form. Indeed, MB has been shown to be hydrolysed rapidly *in vitro* by water-soluble enzymes (hydrolases and esterases) found in both plasma and tissues^(^^41^^)^, and TB is also hydrolysed quickly to butyric acid by esterases/lipases in blood samples, reaching a peak concentration (about 2·2 µm) at 25 min after oral administration of 2 g/kg TB^(^^42^^,^^43^^)^. On the other hand, in a study, by Augustin *et al.*^(^^44^^)^, the radiolabelled TB was found in the caecum and colon of rats, although in lesser amounts than in the small-intestinal walls. However, to our knowledge there are no *in vivo* studies in the literature on the measurement of MB in the gastrointestinal tract. Studies *in vitro*, simulating gastrointestinal conditions, have shown that TB is degraded to MB which survives gastrointestinal conditions (K Schwarzer and J Björk, published conference paper). The pronounced effects on caecal microbiota composition in the present study also indicate that the butyrins are affecting the colonic substrate in some way, e.g. via the bile acids. It is also important to mention that there are effects on SCFA with MB and TB in the caecum, serum and liver. Since metabolic cross-feeding exists between the SCFA, the measurements may be complicated in a starving environment^(^^6^^)^. Thus, it should be considered that instead of using dietary fibre, which is known to increase SCFA formation, the test diets were supplemented with glycerol esters of SCFA, possibly resulting in different SCFA profiles compared with other studies. It is also important to emphasise that the portal butyric acid concentrations tended to be higher in the MB and TB groups than in the HFC group, which may have reached significance at a higher dose or longer exposure.

### Monobutyrin and tributyrin affect liver cholesterol and hepatic gene expression of bile acid enzymes

There was a considerable cholesterol-lowering effect especially by MB, but also by TB. It has been already reported that TB partly decreases serum concentrations of cholesterol and TAG, accompanied with a significant reduction in hepatic TAG accumulation in high fat-fed mice^(^^45^^)^. In the liver, cholesterol is used to generate bile acids, which, in turn, are secreted into the small intestine, stimulating lipid absorption, including cholesterol^(^^46^^)^. The bile acid synthetic pathway is facilitated by *Cyp7a1*, which usually accounts for the pool size of bile acids, followed by the activity of *Cyp8b1* that determines the composition of bile acids. The expression of *Cyp7a1* and *Cyp8b1* was lower in the MB and TB groups, with greater reduced expression of *Cyp8b1* in the MB group, associated with a concomitant decrease in liver cholesterol. As bile acids are necessary for lipid absorption, this finding indicates that the cholesterol-lowering effect could be due to reduced absorption or increased excretion of cholesterol in the intestine, leaving less cholesterol transported back to the liver. In the same manner, it has been reported that cholesterol absorption was reduced, while faecal neutral sterol excretion was increased in *Cyp7a1-*deficient mice^(^^47^^)^. Although contributing to minor changes, it should be mentioned that SCFA, especially butyric acid, may take part in cholesterol metabolism because butyrate has been shown to directly inhibit intestinal lipid transport^(^^48^^)^.

### Liver succinic acid levels are reduced with monobutyrin and tributyrin

The decrease in liver succinic acid, and also the tendency to a decreased succinic acid:butyric acid ratio, observed with MB and TB indicates that this supplementation is attributed to protective effects against high-fat feeding. Succinic acid is traditionally known as a substrate for microbial synthesis of propionate and does not substantially accumulate in the large bowel^(^^6^^)^. The amounts of succinic acid in the liver were positively related to the caecal amounts of propionic acid and also to those in serum (but not to those in the liver) and the amount of liver cholesterol. Likewise, succinic acid concentration can be increased at the expense of butyric acid in the caecum of rats fed high-fat diets and in human subjects having depressed microbial activity in the colon due to treatment with broad-spectrum antibiotics, indicating its harmful effect over time^(^^8^^,^^49^^)^. A number of physiological effects have been connected to succinate and it has been shown to promote *Clostridium difficile* infection in human subjects^(^^9^^,^^49^^)^. Furthermore, elevated succinate in urine and plasma has been reported in animals subjected to metabolic and diabetic disease models, including hypertension^(^^50^^,^^51^^)^. Succinic acid has also been recognised as an LPS-induced inflammatory signal that enhances the production of the inflammatory protein IL-1β in dendritic cells and macrophages^(^^52^^,^^53^^)^. On the other hand, in a recent study succinic acid, but also propionic acid, was demonstrated to be a substrate of intestinal gluconeogenesis and as a consequence had the capacity to improve glucose homeostasis^(^^54^^)^. The observed association between succinic acid and liver cholesterol in this study can be considered as an interesting finding, since succinate has been claimed to increase blood pressure in animals via activation of its receptor GPR91^(^^55^^)^, potentially supporting its role in hypertension-related diseases such as atherosclerosis. Due to its activation on the transcriptional factor hypoxia-inducible factor 1α in both tumours and inflammatory states, succinic acid has been proposed as a signal linking inflammation and cancer^(^^52^^,^^56^^,^^57^^)^. Thus, the role of succinic acid in health and disease states needs further investigation.

### Low-fat diet

The group fed the LF diet had some unexpected results, for example final body weight and body weight gain. This could be due to lower energy content of the LF diet, which was compensated by a higher feed intake to maintain different physiological requirements. Furthermore, when taking the feed intake into account together with the weight gain, feed efficiency ratio was similar between all groups, although it could be expected to be even lower due to the lower energy density of the LF diet. The activity of the rats in different groups may also differ, but no behavioural test was performed and we do not know if the LF group was less active than the other groups or not.

Concerning the SCFA, it can be discussed if a LF diet containing cellulose, which is almost resistant to fermentation, is an optimal control and a description of a normal state for SCFA and thus also the microbiota. It might be better to compare only the high-fat diets. Perhaps it would be more relevant to include a positive control, containing fermentable fibres that are associated with high formation of SCFA. The lack of fermentable fibre and also fat, that indirectly can be a substrate for the microbiota via the bile acids, may be provocative by itself, creating a starving environment in the colon. Such a diet also leads to a high intake of digestible starch, which may change the appetite hormonal profiles resulted in a higher food intake^(^^58^^)^.

The LF reference group used in the study, on the other hand, seems to be appropriate concerning results on lipids. Furthermore, the caecal SCFA was higher and liver succinic acid lower with this group than with the HFC group, which is consistent with other studies.

In conclusion, the results from the present study suggest that supplementation of 5 g/kg of MB and TB into a HFC diet is effective in decreasing liver cholesterol concentration in rats after 3 weeks. This could not clearly be related to any specific SCFA since these were similar whether the butyrins were added or not. Indeed, we observed a down-regulation of hepatic bile acid synthesis genes and a decreased hepatic concentration of succinic acid. However, the mechanisms behind the effects of MB and TB on liver cholesterol need further investigation. The present study also highlights a new role of TB in lipid metabolism, particularly in reduction of liver cholesterol associated with a high-fat diet, and also introduces MB as a promising candidate with similar effects.

## Supplementary material

For supplementary material accompanying this paper visit http://dx.doi.org/10.1017/jns.2017.54. click here to view supplementary material
