# Supplementary material for: Effects of monobutyrin and tributyrin on liver lipid profile, caecal microbiota composition and SCFA in high-fat diet-fed rats
Source: J Nutr Sci. 2017 Oct 11;6:e51. doi: 10.1017/jns.2017.54 (PMC5672331; doi:10.1017/jns.2017.54)
Supplement: Supplementary file 1 [file S2048679017000544sup001.doc]

# Supplementary material

# Methods

**Caecal microbiota***.* DNA was amplified using primers for the target gene 16S rRNA (V3-4 regions: forward 5’-CCTACGGGNGGCWGCAG-3’ and reverse 5’-GACTACHVGGGTATCTAATCC-3’. The preparation of the amplicon library was done according to 16S Metagenomic Sequencing Library Preparation protocol (https://www.illumina.com)

Paired-end sequencing of the library with a read length of 2×300 bp using Miseq V3 reagent kit (the batch produced in 2014) was carried out on a Miseq Instrument (Illumina Inc., San Diego, USA).

Sequencing data (FASTQ format) was analysed using an open-source bioinformatic pipeline, Quantitative Insights into Microbial Ecology (QIIME). Forward and reverse reads were joined and then quality filtering was performed. As a result, a total number of 4 226 985 reads were generated for 29 samples (LF, n=6; HFC, n=5; 0.1MB, n=7; MB, n=6; TB, n=5) with a mean of 145,758 reads per sample (min: 23,278 and max: 312,813). The sequences were grouped into operational taxonomic units (OTUs) at a minimum of 97% similarity, generating 7 OTUs at phylum level and 33 OTUs at genus level. Taxonomy was assigned using the Greengenes database (v.13.8). Alpha rarefaction to estimate alpha diversity was applied to the OTU table at an even depth of 23 000 sequences per sample, thus including all samples in the analysis.

**Gene expression**. Total RNA in freeze-dried liver tissues (approximately 30 mg) was purified using the RNeasy Mini Kit with on-column DNase digestion (Qiagen GmbH, Hilden, Germany), and converted to cDNA using the Thermo Scientific^TM^ RevertAid^TM^ First Strand cDNA Synthesis Kit (Thermo Fisher Scientific). The quantitative real-time PRC was performed using a Bio-Rad T100 Thermal Cycler with SYBR Green SsoAdvanced Universal Supermix (Bio-Rad, California, USA). The amplification protocol was started with an activation at 95°C for 2 min, followed by 40 cycles of 5 s denaturation at 95°C and for 30 s annealing at 60°C. A melt curve analysis was performed to indicate specificity of the PCR products. Gene-specific primers were added in a pre-made PrimePCR plate from Bio-Rad (Solna, Sweden). The ∆∆Ct method was applied to calculate relative mRNA expression, using GAPDH as a reference gene.

# Results

Supplementary Table S1. Composition of ester products (% w/w)

|  | **Mono-butyrin**  **% w/w** | **Di-butyrin % w/w** | **Tri-butyrin % w/w** | **Glycerol % w/w** | **Butyric acid**  **% w/w** |  |  |
| --- | --- | --- | --- | --- | --- | --- | --- |
| **MB** | 46 | 13 | 1.1 | 39 | <1 |  |  |
| **TB** | <1 | 6.1 | 91 | <1 | <1 |  |  |

Supplementary Fig. S1. Ratios of caecal SCFAs in rats fed low-fat (LF) diet, high-fat control (HFC) diet or the HFC diet supplemented with 0.1% monobutyrin (0.1MB), 0.5% monobutyrin (MB) or 0.5% tributyrin (TB) (n=7/group). (A) Acetic-to-propionic acid ratio (B) Acetic-to-butyric acid ratio (C) Acetic-to-propionic-plus-butyric acid ratio. Differences in means between the HFC group and the test groups were determined by one-way ANOVA followed by Dunnett’s test. **P*< 0·05, ***P* <0·01.

Supplementary Table S2. Proportion (%) of acetic-, propionic- and butyric acids in the caecum of rats fed low-fat (LF) diet, high-fat control (HFC) diet or the HFC diet supplemented with 0.1% monobutyrin (0.1MB), 0.5% monobutyrin (MB) or 0.5% tributyrin (TB) (n=7/group)

| SCFA/Groups | LF | | HFC | | 0.1 MB | | MB | | TB | |
| --- | --- | --- | --- | --- | --- | --- | --- | --- | --- | --- |
|  | Mean | SEM | Mean | SEM | Mean | SEM | Mean | SEM | Mean | SEM |
| Acetic acid | 71 | 1.2 | 70 | 1·1 | 71 | 1·0 | 74 | 1·7 | 74 | 1·4 |
| Propionic acid | 16* | 0.6 | 13 | 0·5 | 15 | 0·6 | 11* | 0·4 | 11* | 0·7 |
| Butyric acid | 10 | 0.5 | 11 | 0·6 | 11 | 0·8 | 13 | 0·7 | 12 | 1·1 |

Mean values were significantly different from the HFC group: **P*<0·05.
